# Transport-exclusion pharmacology to localize lactate dehydrogenase activity within cells

**DOI:** 10.1186/s40170-018-0192-5

**Published:** 2018-12-12

**Authors:** Xiangfeng Niu, Ying-Jr Chen, Peter A. Crawford, Gary J. Patti

**Affiliations:** 1 Department of Chemistry, Washington University, St. Louis, USA; 2 Division of Molecular Medicine, Department of Medicine, University of Minnesota, Minneapolis, USA; 3 Department of Biochemistry, Molecular Biology, and Biophysics, University of Minnesota, Minneapolis, USA; 4 Department of Medicine, Washington University School of Medicine, St. Louis, USA

**Keywords:** Lactate, Lactate dehydrogenase, Transport-exclusion pharmacology, Redox balance

## Abstract

**Background:**

Recent *in vitro* and *in vivo* work has shown that lactate provides an important source of carbon for metabolic reactions in cancer cell mitochondria. An interesting question is whether lactate is oxidized by lactate dehydrogenase (LDH) in the cytosol and/or in mitochondria. Since metabolic processes in the cytosol and mitochondria are affected by redox balance, the location of LDH may have important regulatory implications in cancer metabolism.

**Methods:**

Within most mammalian cells, metabolic processes are physically separated by membrane-bound compartments. Our general understanding of this spatial organization and its role in cellular function, however, suffers from the limited number of techniques to localize enzymatic activities within a cell. Here, we describe an approach to assess metabolic compartmentalization by monitoring the activity of pharmacological inhibitors that cannot be transported into specific cellular compartments.

**Results:**

Oxamate, which chemically resembles pyruvate, is transported into mitochondria and inhibits LDH activity in purified mitochondria. GSK-2837808A, in contrast, is a competitive inhibitor of NAD, which cannot cross the inner mitochondrial membrane. GSK-2837808A did not inhibit the LDH activity of intact mitochondria, but GSK-2837808A did inhibit LDH activity after the inner mitochondrial membrane was disrupted.

**Conclusions:**

Our results are consistent with some mitochondrial LDH that is accessible to oxamate, but inaccessible to GSK-2837808A until mitochondria are homogenized. This strategy of using inhibitors with selective access to subcellular compartments, which we refer to as transport-exclusion pharmacology, is broadly applicable to localize other metabolic reactions within cells.

**Electronic supplementary material:**

The online version of this article (10.1186/s40170-018-0192-5) contains supplementary material, which is available to authorized users.

## Background

Most mammalian cells contain organelles that are bounded by lipid membranes. The chemical reactions occurring in each of these compartments are sequestered from the rest of the cell, thereby providing an opportunity to specialize metabolism in support of specific organelle functions. Some examples include generating harmful metabolic byproducts in organelles where they can be neutralized (such as hydrogen peroxide in peroxisomes), adjusting chemical concentrations to drive reactions in a direction that they may not proceed in other parts of the cell (such as using a proton gradient to fuel ATP synthesis in mitochondria), and harboring anabolic and catabolic reactions in different compartments to limit unproductive futile cycling (such as fatty acid synthesis in the cytosol and fatty acid oxidation in mitochondria) [1, 2].

Despite increasing evidence that metabolic compartmentalization is essential to various cellular functions, the spatial organization of metabolism within a cell remains poorly understood due to the technical challenges of measuring subcellular location. In a typical metabolomic experiment, cell lysates are analyzed and the results therefore only provide average concentrations of metabolites from the entire cell. Although organelles can be efficiently purified for metabolic evaluation, co-purified contaminates are a considerable challenge [3]. Metabolite interactions with the outer membrane leaflet or its associated proteins, for example, can complicate data analysis. For mitochondria, which are the focus of the current work, the intermembrane space creates additional difficulties. Proteomic analyses suffer from the same problems [4]. High-resolution microscopy can be applied to image proteins within a cell, but this does not reflect protein activity. Functional assays from purified mitochondria can be insightful; however, it is difficult to confirm that protein activity occurs within the mitochondrial matrix. Further, an incomplete understanding of mitochondrial carrier systems has limited our ability to study compartmentalization by manipulating metabolite transport [5]. Thus, strategies to localize metabolic transformations within subcellular compartments such as mitochondria are highly needed.

In this work, we were specifically interested in localizing the enzyme lactate dehydrogenase (LDH) within cells. Recent studies have shown that some cancer cells use lactate *in vitro* and *in vivo* as a primary carbon source for metabolic pathways in mitochondria, such as the tricarboxylic acid (TCA) cycle [6–8]. LDH is required to incorporate lactate carbon into TCA cycle intermediates. An interesting question is whether this LDH activity occurs in the cytosol and/or in mitochondria. When oxidizing lactate to pyruvate, LDH simultaneously reduces NAD^+^ to NADH. Neither NAD^+^ nor NADH can cross the inner mitochondrial membrane, and the ratio of NAD^+^ to NADH modulates numerous biological processes in both the cytosol and mitochondria. Thus, the location of LDH may selectively influence redox balance within subcellular compartments and therefore have important regulatory implications in cancer metabolism [9].

## Methods

### Cell culture and drug treatments

Unless otherwise noted, cells were cultured in high-glucose Dulbecco’s Modified Eagle Medium (DMEM, 4.5 g/L D-glucose) (Life Technologies) containing 10% Fetal Bovine Serum (FBS) (Life Technologies) and 1% penicillin/streptomycin (Life Technologies) at 37 °C with 5% CO_2_. In each drug experiment, either oxamate or GSK-2837808A (3-[[3-[(Cyclopropylamino) sulfonyl]-7-(2,4-dimethoxy-5-pyrimidinyl)-4-quinolinyl] amino]-5-(3,5-difluorophenoxy) benzoic acid, TOCRIS) was added into the assay buffer. To account for effects of DMSO, DMSO was added to the assay buffer in all experiments (including oxamate conditions and vehicle conditions). The final concentration of DMSO was 1%, unless otherwise stated. Three biological replicates were used for each condition tested.

### Lactate production assay

Approximately 7 × 10^5^ HeLa cells were seeded in a 12-well plate and allowed to attach overnight. Cells were then washed and supplemented with FBS-free, low-glucose media (1 g/L D-glucose) and treated with oxamate, GSK-2837808A, or DMSO alone (vehicle). After 6 h, the culture media were collected and extracted as described previously and detailed below [10]. Samples were analyzed by liquid chromatography/mass spectrometry (LC/MS) in negative ion mode with a triple quadrupole mass spectrometer (6460, Agilent Technologies). Samples were separated with a Luna Aminopropyl column (3 μm, 150 mm × 1.0 mm I.D., Phenomenex) coupled to an Agilent 1260 LC system. A flow rate of 50 μL/min was used. The mobile phases and linear gradient were A = 95% water, 5% acetonitrile (ACN), 20 mM ammonium hydroxide (NH_4_OH), 20 mM ammonium acetate (NH_4_Ac); B = 100% ACN; 85% B from 0 to 3 min, 85% to 50% B from 3 to 7 min, 50% to 5% B from 7 to 11 min, and 5% B from 11 to 13 min.

### Purification of mitochondria

Mitochondria were purified as described previously [6]. Briefly, cells were harvested, pelleted, and re-suspended in cold mitochondrial isolation media (MIM) (300 mM sucrose, 10 mM HEPES, 0.2 mM EDTA, and 1 mg/mL bovine serum albumin (BSA), pH 7.4) and then homogenized with a glass-Teflon potter. Next, samples were centrifuged at 700×*g* (4 °C) for 7 min to separate mitochondria from the remaining cellular material. The supernatant was decanted after centrifugation and set aside. The remaining pellets were homogenized again in MIM to recover more mitochondria. The supernatant was then pooled with the supernatant from above and centrifuged at 10,000×*g* (4 °C) for 10 min to obtain mitochondrial pellets. Mitochondrial pellets were washed and quantified by performing a Bradford assay, unless otherwise noted.

### LDH activity assay

LDH activity was assessed in a 96-well plate. First, mitochondria were purified from ~ 6 × 10^7^ HeLa cells as above. Mitochondrial pellets were then lysed with 1% triton X-100/50 mM Tris (pH 7.4). The mitochondrial lysates were treated with oxamate, GSK-2837808A, or DMSO alone (vehicle). The 1% triton X-100/50 mM Tris solution was used as a negative control (blank). A standard mixture was prepared containing phenazine methosulphate (360 μg/mL), p-iodonitrotetrazolium violet (1.3 mg/mL), and NAD^+^ (340 μg/mL). A 50 μL aliquot of the standard mixture, 200 mM Tris (pH 8), and 50 mM lactate were added to each well before adding 50 μL of sample (38 μg of mitochondrial protein/well). The final concentration of DMSO was 0.4% in all three conditions. The kinetic assay was run at 490 nm with a Cytation 5 microplate reader (BioTek), and LDH activity was determined by the maximum slope.

### Labeling whole cells with U-^13^C lactate

HeLa cells were grown to ~ 35% confluency in 100-mm culture dishes. The culture media were then changed to fresh low-glucose (5 mM) media supplemented with 3 mM uniformly ^13^C-labeled lactate (U-^13^C lactate, Cambridge Isotope Laboratories). Cells were treated with 50 mM oxamate, 75 µM GSK-2837808A, or DMSO alone (vehicle) for 24 h. The final concentration of DMSO was 0.3% in all three conditions. After 24 h, cells were washed with phosphate-buffered saline (PBS) and HPLC-grade water, quenched with 1 mL cold HPLC-grade methanol, scraped from the plate, and pelleted. Pellets were dried on a SpeedVac (Thermo Fisher Scientific) and subsequently lyophilized (Labconco). Dried samples were weighed and extracted by using the protocol described below [10]. Experiments were performed with *n* = 3 cultures per sample group.

### Oxygen consumption rate

Respiration of intact mitochondria was measured with an XFp analyzer (Seahorse Bioscience) or a high-resolution OROBOROS Oxygraph-2k respirometer (Oroboros Instruments). For the Seahorse experiments, after purifying mitochondria from ~ 2 × 10^7^ HeLa cells, approximately 8 μg of mitochondrial pellets were re-suspended in cold mitochondria assay solution (MAS, 70 mM sucrose, 220 mM mannitol, 10 mM KH_2_PO_4_, 5 mM MgCl_2_, 2 mM HEPES, 1 mM EGTA, and 0.2% (*w*/*v*) fatty acid-free BSA, pH 7.2) with 10 mM lactate and 5 mM malate. Before measuring respiration, mitochondria were brought to room temperature. ADP (4 mM) was added to induce respiration. The oxygen consumption rate (OCR) of HeLa mitochondria was monitored under three different conditions: oxamate, GSK-2837808A, or DMSO alone (vehicle). To remove background contributions, the OCR value before the addition of ADP was subtracted from the OCR value after the addition of ADP (Fig. 3b). For assessing mitochondrial function, no drugs or DMSO were added. Sample sizes were used that produced OCR numbers within the recommended range of the vendor. For the Oroboros experiments, mitochondrial respiration media were used. Approximately the same number of HeLa cell mitochondria (300 μg mitochondrial protein) was added to each chamber followed by metabolic substrates and inhibitors.

### Labeling whole cells with 2-^2^H lactate prior to mitochondrial purification

Cells were cultured in a T-150 flask until reaching 90% confluency. Cells were then transferred to glucose-free media for 4 h. After 4 h, cells were supplemented with 10 mM 2-^2^H lactate for 45 min prior to being washed, harvested, and pelleted. For mitochondrial purification, the cell pellets were re-suspended in 500 μL of cold MIM with 100 mM oxamate. The isolated mitochondrial pellets were lyophilized and subsequently treated with a methanol/acetonitrile/water (2:2:1) solution prior to being reconstituted in 40 μL acetonitrile/water (1:1) per milligram dry weight. LC/MS analysis was performed as described below.

### Labeling purified mitochondria with U-^13^C lactate

Approximately 2 × 10^8^ HeLa cells were harvested at 90% confluence. Mitochondria were purified as above. Purified mitochondria were split into wells (170 μg of mitochondrial protein/well) and incubated in 1 mL MAS buffer with 5 mM malate and 5 mM lactate. Samples were treated with oxamate, GSK-2837808A, or DMSO alone (vehicle) for 10 min. The final concentration of DMSO was 0.3% in all three conditions. After 10 min, 10 mM U-^13^C lactate was added to the MAS buffer for 20 min before harvesting. Mitochondrial pellets were washed, collected, and snap frozen in liquid nitrogen prior to extraction.

### Metabolite extraction and LC/MS analysis

Cell pellets or purified mitochondria were extracted and analyzed by LC/MS as described before [6, 10]. Cell pellets were treated with a methanol/acetonitrile/water (2:2:1) solution and reconstituted in 40 μL acetonitrile/water (1:1) per milligram dry weight. Mitochondrial pellets were treated with a methanol/acetonitrile/water (2:2:1) solution and reconstituted in 50 μL acetonitrile/water (1:1) per 170 μg of mitochondrial protein, unless otherwise noted. Samples were separated with a Luna Aminopropyl column (3 μm, 150 mm × 1.0 mm I.D., Phenomenex) coupled to a Dionex UltiMate® 3000 RSLCnano LC system. MS detection was performed on a Thermo Q Exactive Plus mass spectrometer (Thermo Fischer Scientific) in negative ion mode at 140,000 resolving power. The column was used in hydrophilic interaction mode with a flow rate of 50 μL/min. The following mobile phases and linear gradient were used: A = 95% water, 5% ACN, 20 mM NH_4_OH, 20 mM NH_4_Ac; B = 95% ACN, 5% water; 100% B from 0 to 3 min, 100% B to 0% B from 3 to 40 min, and 0% B from 40 to 45 min.

### Statistical analysis

Data are reported as means ± SD. Dataset comparisons were performed with a Student’s unpaired, two-tailed *t* test.

## Results

**Fig. 1:**
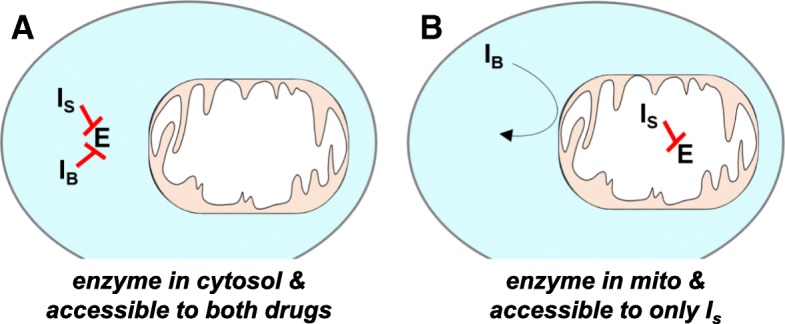
Transport-exclusion pharmacology to localize enzyme activity within a cell. **a** When the enzyme is localized to the cytosol, both inhibitors block activity. **b** When the enzyme is localized to mitochondria, only *I*_S_ blocks activity. *E* represents the enzyme of interest localized in cytosol or mitochondria. *I*_S_ represents an inhibitor of E that is transported into mitochondria. *I*_B_ represents an inhibitor of *E* that cannot be transported into mitochondria

Here, we describe an approach to localize metabolic reactions to the mitochondrial matrix by exploiting the limited permeability of the inner mitochondrial membrane to small molecule inhibitors. The method, which we refer to as transport-exclusion pharmacology, assumes that the activity of enzymes in the mitochondrial matrix is only affected by exogenously delivered inhibitors that cross the inner mitochondrial membrane. When an inhibitor without access to the mitochondrial matrix does not affect enzymatic activity in whole-cell analyses, this suggests that the enzyme is exclusively localized to the matrix (Fig. 1). When whole-cell experiments show reduced enzymatic activity, in contrast, it indicates that at least some enzyme has an extramitochondrial location. To identify cases of redundancy where enzymes (e.g., LDH) are located in both the mitochondrial matrix as well as other cellular locations with potentially differential access to inhibitors, the same experiment can be repeated on isolated mitochondria. When testing isolated mitochondria with inhibitors that do not have access to the matrix and enzyme activity is unaffected, this indicates enzyme localization to the matrix. All results can be validated by using homogenized mitochondria, which allows inhibitors to access matrix-localized proteins independent of their transport properties. We applied this strategy to localize LDH in HeLa cells here because we already evaluated the spatial organization of LDH in these cells with other methods previously [6].

Functional LDH is a homo- or heterotetramer made up of LDHA and LDHB subunits [11]. We considered two compounds (oxamate and GSK-29837808A) known to inhibit both LDHA and LDHB subunits at the concentrations we used [12–16]. We note that because our inhibition experiments are not specific to enzyme subtype, we cannot distinguish between LDHA and LDHB in the analyses. It has been shown previously, however, that LDHB is concentrated in HeLa cell mitochondria [6].

**Fig. 2:**
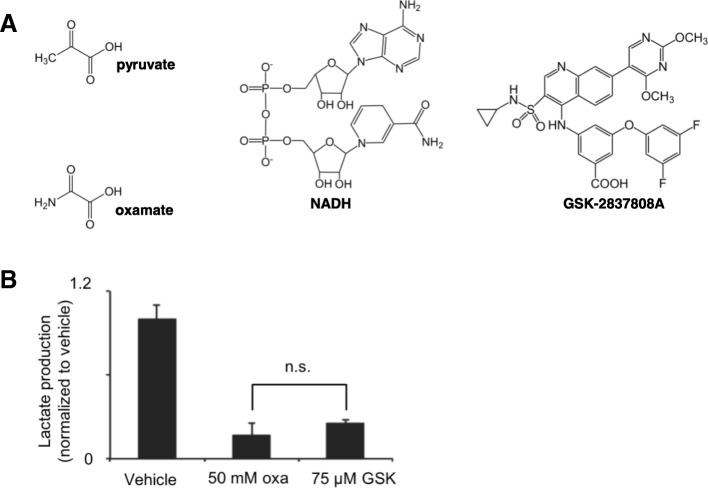
Oxamate and GSK-2837808A inhibit LDH activity in HeLa cells. **a** Oxamate resembles pyruvate in structure and competes with pyruvate for LDH binding. GSK-2837808A competes with NAD for LDH binding. **b** Different concentrations of oxamate (oxa) and GSK-2837808A (GSK) achieved a similar level of LDH inhibition, as determined by measuring lactate excreted to the HeLa extracellular media after drug treatment. All data shown are averages from groups of *n* = 3. n.s., no statistical difference

Oxamate is an isoelectric and isosteric analogue of pyruvate, having a dissociation constant with LDH/NADH that is close to the Michaelis-Menten constant of pyruvate [17]. Additionally, oxamate is transported into mitochondria by the mitochondrial pyruvate carrier [18]. GSK-29837808A, on the other hand, is a competitive inhibitor of NAD, which cannot cross the inner mitochondrial membrane [12]. When given to intact HeLa cells, both compounds inhibited LDH activity as measured by the amount of lactate excreted into the media (Fig. 2). Consistent with previous reports, we found that micromolar concentrations of GSK-29837808A achieved the same level of LDH inhibition as millimolar concentrations of oxamate [13]. These results show that, as expected, LDH is not exclusively localized to HeLa cell mitochondria.

**Fig. 3:**
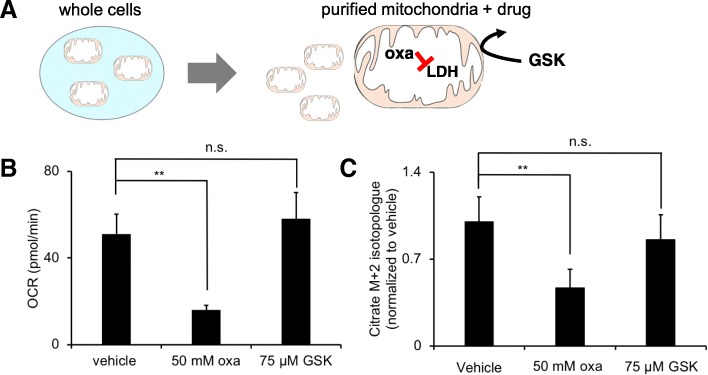
Only oxamate significantly inhibits LDH activity when drugs are given to purified mitochondria from HeLa cells. **a** Mitochondria were first purified from HeLa cells and then given oxamate (oxa) or GSK-2837808A (GSK). **b** Oxygen consumption rate (OCR) from purified mitochondria provided lactate as a respiratory substrate. Each condition is compared to a vehicle control having an identical preparation, but without drug treatment. **c** M + 2 labeling in citrate from purified mitochondria provided U-^13^C lactate as a respiratory substrate. The M + 2 isotopologue is significantly decreased due to oxamate, but not significantly different from the vehicle control due to GSK-2837808A treatment. All data shown are averages from groups of *n* = 3. **p < 0.01; n.s., no statistical difference

We next wished to consider the possibility that LDH is present in both the cytosol and mitochondria, as has been suggested previously [19–23]. To evaluate mitochondrial metabolism specifically, we purified intact mitochondria from HeLa cells and verified their integrity and metabolic function. We determined the respiratory control ratio (state 3/state 4) of purified HeLa cell mitochondria to be > 8. We also confirmed that our purified HeLa cell mitochondria responded to the addition of various substrates and inhibitors as expected (Additional files 1 and 2: Figures S1 and S2). After purifying intact mitochondria from HeLa cells, we treated them with oxamate or GSK-29837808A and then assessed LDH activity in either of two ways. First, we measured the ADP-supported OCR of vehicle controls and compared them to purified mitochondria treated with drug (Fig. 3a). We used a concentration of 50 mM for oxamate and 75 μM for GSK-29837808A, since these concentrations were determined to have comparable effects on LDH activity in HeLa cells (Fig. 2). Importantly, purified mitochondria were only provided malate (5 mM) and lactate (10 mM) as carbon sources in these experiments. We note that purified HeLa mitochondria do not respire well when only provided malate or lactate alone, presumably because they need a source of oxaloacetate and acetyl-CoA for citrate synthase. Thus, under our experimental conditions, respiration can only be supported by LDH oxidation of lactate. Interestingly, we found oxamate reduced the OCR by ~ 70% while GSK-29837808A had no statistically significant effect relative to vehicle controls (Fig. 3b).

As a second method to assess LDH activity in purified HeLa cell mitochondria, we also used stable isotope tracers and LC/MS. After purifying HeLa mitochondria, we incubated them in the same buffer as above, but we added U-^13^C lactate for 20 min. We then used the incorporation of two ^13^C labels into citrate as an indicator of LDH activity. The M + 2 isotopologue of citrate results from the sequential actions of LDH, the pyruvate dehydrogenase complex, and citrate synthase. Consistent with the OCR data, we found that oxamate reduced citrate labeling while GSK-29837808A treatment led to no statistically significant change relative to vehicle controls (Fig. 3c). Together, these data are consistent with LDH localization to the mitochondrial matrix, where it is accessible to oxamate but not GSK-29837808A (rather than localization to the outer membrane or intermembrane space).

**Fig. 4:**
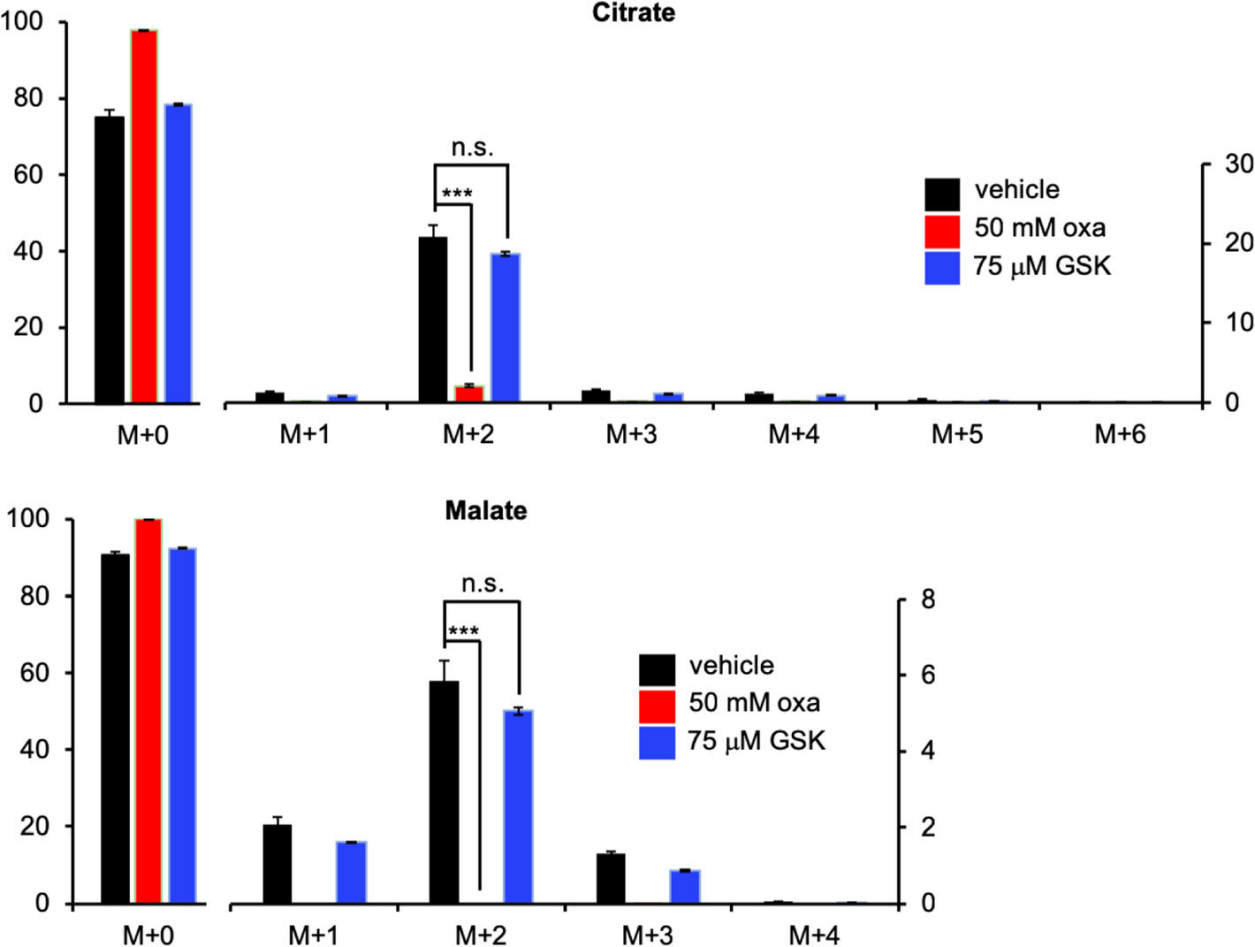
TCA cycle labeling from U-^13^C lactate is decreased when whole cells are treated with oxamate, but not when whole cells are treated with GSK-2837808A. These results are consistent with oxamate (oxa) inhibiting cytosolic and mitochondrial LDH, but with GSK-2837808A (GSK) only inhibiting cytosolic LDH. HeLa cells were labeled with U-^13^C lactate for 24 h prior to analysis of whole-cell extracts by LC/MS. Data shown are averages from groups of *n* = 3. ***p < 0.001. n.s., no statistical significance. Left *y* axis shows labeling from 0 to 100%. Right *y* axis shows a zoomed-in scale for ^13^C isotopologues (inset)

Having established that U-^13^C lactate labeled TCA cycle intermediates differently when purified mitochondria were treated with oxamate versus GSK-29837808A, we sought to repeat the experiment with whole cells. We cultured HeLa cells in media containing 5 mM glucose and 3 mM U-^13^C lactate for 24 h. For the duration of labeling, samples were treated either with 50 mM oxamate, 75 μM GSK-29837808A, or vehicle control (DMSO alone). After 24 h, intact cells were harvested and extracted for LC/MS analysis. Strikingly, we observed only minimal labeling of TCA cycle intermediates in cells treated with oxamate (Fig. 4). In contrast, labeling of cells treated with GSK-29837808A was not significantly different than vehicle controls. Notably, the labeling patterns we observed here for whole cells were consistent with the results obtained when the same experiment was performed on purified mitochondria (Fig. 3c). As described above (Fig. 2b), oxamate and GSK-29837808A both significantly decreased lactate production in whole cells when administered at the same concentration as in Fig. 4. Taken together, these results are consistent with both oxamate and GSK-29837808A decreasing the reduction of pyruvate to lactate by inhibiting cytosolic LDH, but only oxamate having access to inhibit mitochondrial LDH that is used for lactate oxidation. We note that the level of AMP was increased in HeLa cells treated with oxamate but not HeLa cells treated with GSK-29837808A, suggesting that mitochondrial LDH activity may influence cellular energy status (Additional file 3: Figure S3) [24].

**Fig. 5:**
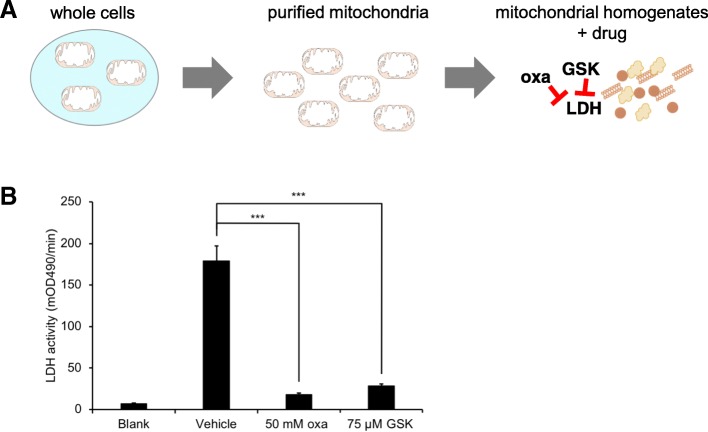
Both oxamate and GSK-2837808A significantly inhibit LDH activity in HeLa cell mitochondrial homogenates. **a** Mitochondria were first purified from HeLa cells and then homogenized before treating with oxamate (oxa) or GSK-2837808A (GSK). **b** LDH activity was measured by using a colorimetric assay. Both drugs significantly inhibited LDH activity compared to vehicle control. Data shown are averages from groups of *n* = 3. ***p < 0.001

To validate that 75 μM GSK-29837808A can effectively inhibit mitochondrial LDH, we homogenized purified mitochondria and assessed LDH activity after drug treatment by using a colorimetric assay. Compared to vehicle control, both 50 mM oxamate and 75 μM GSK-29837808A decreased LDH activity by more than five-fold (Fig. 5). The decrease in LDH activity was less with a lower concentration of oxamate, but the results were comparable (Additional file 4: Figure S4). These data indicate that GSK-29837808A can only access LDH after the inner mitochondrial membrane has been disrupted, which suggests that at least some LDH enzyme is localized to the HeLa mitochondrial matrix. Although we have focused on the metabolism of lactate by HeLa cells in the current study, additional data support that oxidation of lactate by mitochondria is relevant to other cancer cell lines (e.g., Hep G2 and H460) as well (Additional file 5: Figure S5).

## Discussion

Increasing evidence supports that lactate is not only a prominent fuel in cancer cells, but also a major source of carbon for anabolic processes such as lipid synthesis [6–8, 25]. Utilization of lactate in this capacity requires that lactate be oxidized by LDH, which could potentially occur in the cytosolic and/or mitochondrial compartments of a cancer cell. While the difference between cytosolic and mitochondrial LDH activity only changes the location of two electrons, the regulatory implications of a compartmentalized shift in redox homeostasis are potentially significant. Many metabolic enzymes are regulated by the ratio of NAD^+^ to NADH, such as glyceraldehyde 3-phosphate dehydrogenase in the cytosol and isocitrate dehydrogenase 3 in mitochondria [26]. The site of LDH activity may therefore have an important effect on metabolic fluxes. Oxidation of lactate by cytosolic LDH, for example, may slow glycolytic flux and glucose consumption, while oxidation of lactate by mitochondrial LDH may promote lipid synthesis via the accumulation of citrate [6, 9].

Although the possibility of a mitochondrial LDH has been considered for several decades, disagreements persist about its precise location and its biochemical role, as has recently been reviewed in detail [27–29]. Progress in the field has been complicated by the technical limitations of localizing lactate metabolism within different subcellular compartments. Mitochondrial LDH cannot be assessed by tracing labels from ^13^C-lactate because of cytosolic lactate-pyruvate exchange [30]. Imaging approaches have generally provided limited resolution to localize LDH to the mitochondrial matrix. Functional assays examining whether mammalian mitochondrial preparations oxidize lactate have provided mixed results. Thus, alternative experimental approaches to provide additional information about mitochondrial lactate metabolism are needed.

We have described a strategy that exploits unique mitochondrial transport properties of different small-molecule inhibitors (oxamate and GSK-29837808A) to assess LDH location within cells. Interestingly, when evaluating intact purified mitochondria, oxamate decreased LDH activity but GSK-29837808A did not. Yet, both drugs had similar effects on LDH activity once mitochondria were disrupted, indicating differential access to LDH in previous experiments. Even though we have applied the approach to LDH here, the same approach can be broadly applied to investigate the spatial location of other enzymes. These data will be highly complementary to metabolomic and proteomic experiments performed on purified mitochondria and may provide key insights as we seek to understand the ways in which mitochondria are functionally integrated within the cell. We note that employing transport-exclusion pharmacology requires having inhibitors with unique biochemical properties to mediate subcellular distribution, which are not available for many enzymes. However, we are optimistic that the rapidly growing interest in mitochondrial biology will inspire the development of such drugs not only for these types of experiments but also for their potential therapeutic significance in selectively targeting disease processes within specific subcellular compartments.

## Conclusions

Mitochondria isolated from HeLa cells have the capacity to oxidize lactate, suggesting the presence of a mitochondrial LDH. Of particular interest, however, is the location of the enzyme within mitochondria. Its association with the outer mitochondrial membrane, the intermembrane space, or the outer leaflet of the inner mitochondrial membrane affects cytosolic redox balance. Its association with the inner leaflet of the inner membrane or the matrix, in contrast, affects mitochondrial redox balance. Intact purified mitochondria given oxamate, a competitive inhibitor of pyruvate, show decreased LDH activity. GSK-29837808A, which is a competitive inhibitor of NAD, only affected LDH activity in mitochondrial homogenates when the inner mitochondrial membrane had been disrupted. These data are consistent with some mitochondrial LDH that is accessible to oxamate but not GSK-29837808A. Our approach of using small-molecule inhibitors with different mitochondrial transport properties to localize protein activity is broadly applicable to the study of other enzymes.

## Additional files

Additional file 1: **Figure S1.** OCR data from purified HeLa cell mitochondria. State 1 data are from purified mitochondria alone. State 2 data are from purified mitochondria incubated with 10 mM lactate and 5 mM malate, without ADP. State 3 data are from purified mitochondria incubated with 10 mM lactate, 5 mM malate, and 4 mM ADP. Data shown are averages from groups of *n* = 3. **p < 0.01 and ***p < 0.001. (PDF 18 kb)

Additional file 2: **Figure S2**. Respiration of purified mitochondria from HeLa cells. OCR was measured in response to the addition of mitochondria (mito), substrates (pyruvate/malate, pyr/mal; ADP; succinate, suc), inhibitors (rotenone, Rot; oligomycin, Oligo; antimycin A, AntA), an uncoupler (FCCP), and cytochrome *c* (Cyt c). (PDF 55 kb)

Additional file 3: **Figure S3.** AMP levels are increased after treating HeLa cells with oxamate (oxa) for 24 h but not after treating them with GSK-2837808A (GSK) for 24 h. No significant changes in the levels of ATP or ADP were found. All data shown are averages from groups of *n* = 3. *p < 0.05; n.s., no statistical significance. (PDF 42 kb)

Additional file 4: **Figure S4.** Response of HeLa cells to different concentrations of oxamate. **a** HeLa cell mitochondrial lysates were treated with oxamate, and LDH activity was measured by using a colorimetric assay. **b** HeLa cell proliferation was measured after treatment with oxamate for 24 h. No DMSO was added to the samples. Data shown are averages from groups of *n* = 3. ***p < 0.001; n.s., no statistical significance. (PDF 60 kb)

Additional file 5: **Figure S5.** Deuterium labels from 2-^2^H lactate enter Hep G2 and H460 mitochondria. **a** Lactate can be exchanged with pyruvate in the cytosol or lactate can be transported into mitochondria. We sought to distinguish between these two possibilities by labeling cells with 2-^2^H lactate. We then used LC/MS to localize 2-^2^H lactate to mitochondria. **b**, **c** Mass spectra from mitochondria purified from cells cultured for 45 min in 2-^2^H lactate enriched media. **b** Purified mitochondria from Hep G2, a human liver cancer cell line. **c** Purified mitochondria from H460, a human lung cancer cell line. These results support a pool of transported lactate in mitochondria. (PDF 145 kb)
