# Supplementary material for: Transport-exclusion pharmacology to localize lactate dehydrogenase activity within cells
Source: Cancer Metab. 2018 Dec 12;6:19. doi: 10.1186/s40170-018-0192-5 (PMC6290536; doi:10.1186/s40170-018-0192-5)
Supplement: Supplementary file 1 — Figure S1. OCR data from purified HeLa cell mitochondria. State 1 data are from purified mitochondria alone. State 2 data are from purified mitochondria incubated with 10 mM lactate and 5 mM malate, without ADP. State 3 data are from purified mitochondria incubated with 10 mM lactate, 5 mM malate, and 4 mM ADP. Data shown are averages from groups of n = 3. **p < 0.01 and ***p < 0.001. (PDF 18 kb) [file 40170_2018_192_MOESM1_ESM.pdf]

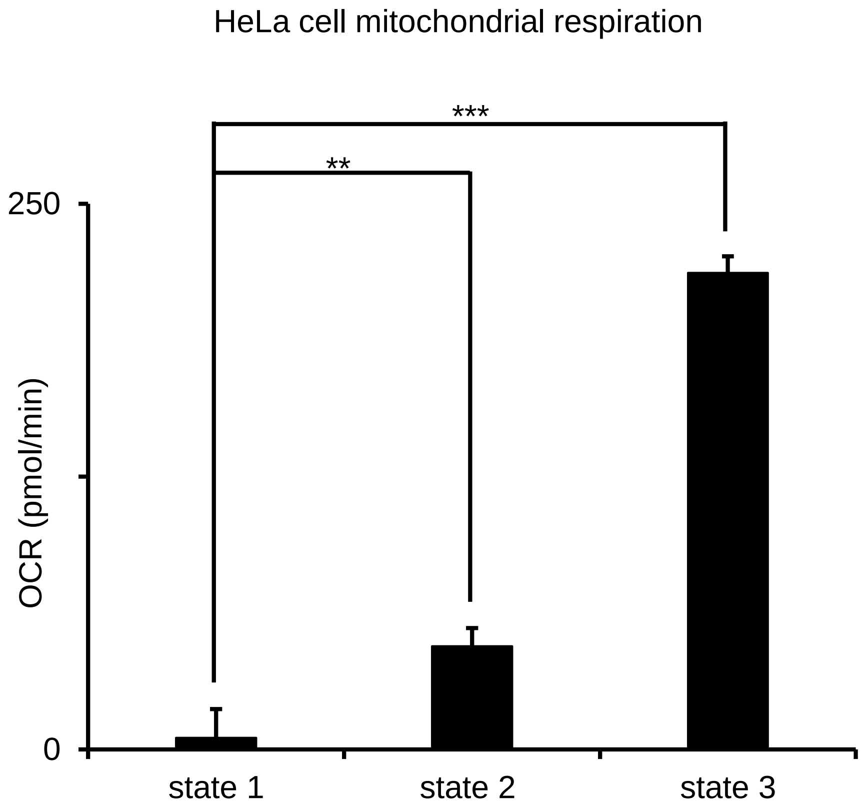

Figure S1: OCR data from purified HeLa cell mitochondria. State 1 data are from purified mitochondria alone. State 2 data are from purified mitochondria incubated with 10 mM lactate and 5 mM malate, without ADP. State 3 data are from purified mitochondria incubated with 10 mM lactate, 5 mM malate, and 4 mM ADP. Data shown are averages from groups of n=3. ** $p<0.01$ and ***$p<0.001$.
