# Supplementary material for: Transport-exclusion pharmacology to localize lactate dehydrogenase activity within cells
Source: Cancer Metab. 2018 Dec 12;6:19. doi: 10.1186/s40170-018-0192-5 (PMC6290536; doi:10.1186/s40170-018-0192-5)
Supplement: Supplementary file 2 — Figure S2. Respiration of purified mitochondria from HeLa cells. OCR was measured in response to the addition of mitochondria (mito), substrates (pyruvate/malate, pyr/mal; ADP; succinate, suc), inhibitors (rotenone, Rot; oligomycin, Oligo; antimycin A, AntA), an uncoupler (FCCP), and cytochrome c (Cyt c). (PDF 55 kb) [file 40170_2018_192_MOESM2_ESM.pdf]

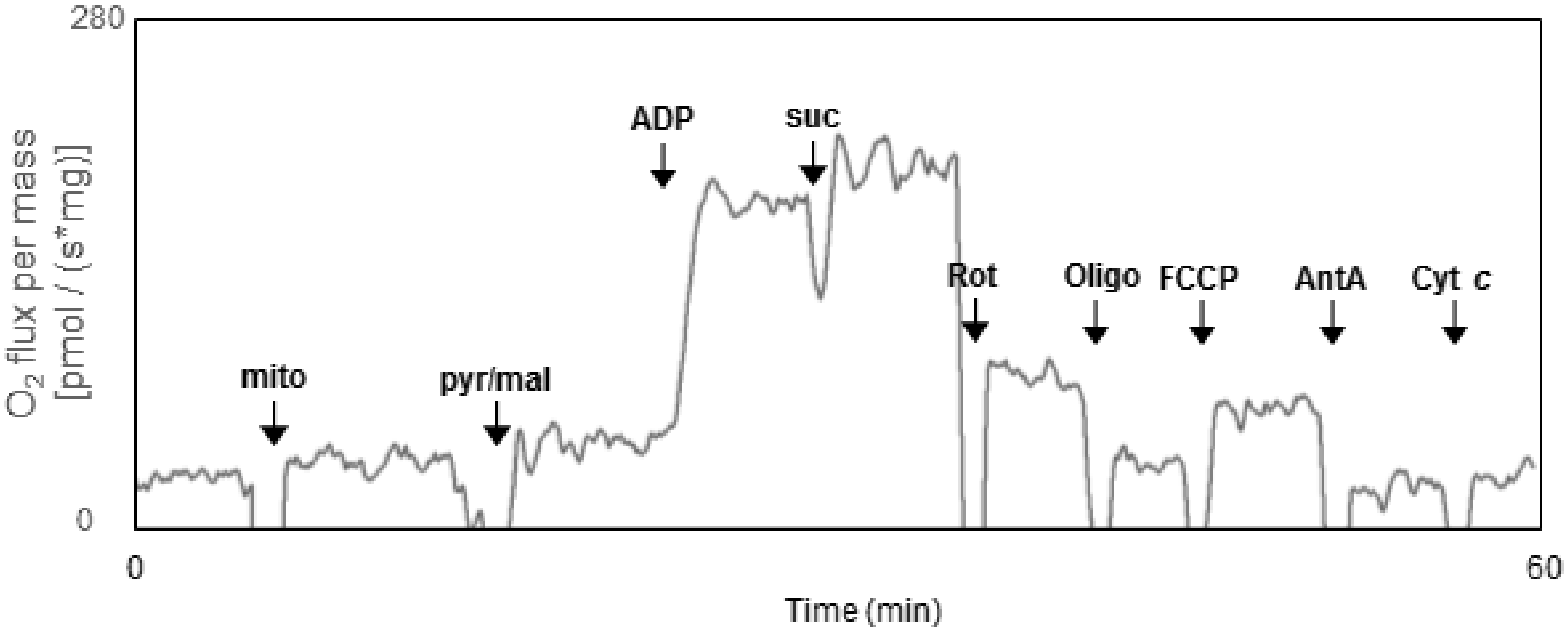

Figure S2: Respiration of purified mitochondria from HeLa cells.
Oxygen consumption rate was measured in response to the addition of mitochondria (mito), substrates (pyruvate/malate, pyr/mal; ADP; succinate, suc), inhibitors (rotenone, Rot; oligomycin, Oligo; antimycin A, AntA), an uncoupler (FCCP), and cytochrome *c* (Cyt c).
