# Supplementary material for: Transport-exclusion pharmacology to localize lactate dehydrogenase activity within cells
Source: Cancer Metab. 2018 Dec 12;6:19. doi: 10.1186/s40170-018-0192-5 (PMC6290536; doi:10.1186/s40170-018-0192-5)
Supplement: Supplementary file 3 — Figure S3. AMP levels are increased after treating HeLa cells with oxamate (oxa) for 24 h but not after treating them with GSK-2837808A (GSK) for 24 h. No significant changes in the levels of ATP or ADP were found. All data shown are averages from groups of n = 3. *p < 0.05; n.s., no statistical significance. (PDF 42 kb) [file 40170_2018_192_MOESM3_ESM.pdf]

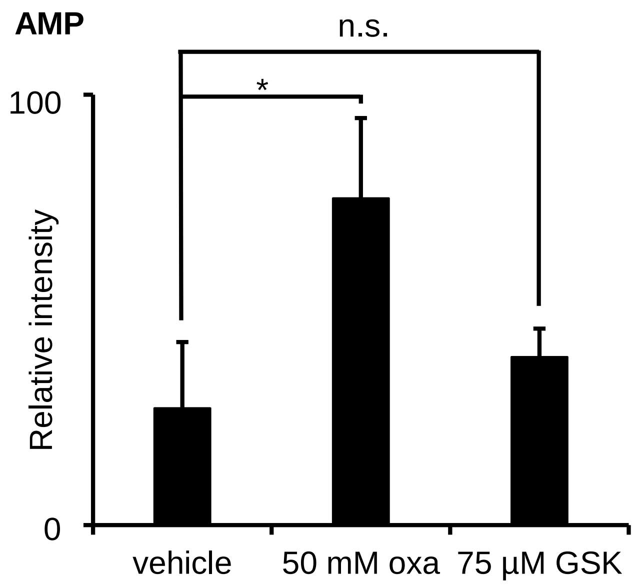

Figure S3: AMP levels are increased after treating HeLa cells with oxamate (oxa) for 24 h but not after treating them with GSK-2837808A (GSK) for 24 h. No significant changes in the levels of ATP or ADP were found. All data shown are averages from groups of n=3. * $p<0.05$; n.s., no statistical significance.
