# Supplementary material for: Transport-exclusion pharmacology to localize lactate dehydrogenase activity within cells
Source: Cancer Metab. 2018 Dec 12;6:19. doi: 10.1186/s40170-018-0192-5 (PMC6290536; doi:10.1186/s40170-018-0192-5)
Supplement: Supplementary file 4 — Figure S4. Response of HeLa cells to different concentrations of oxamate. a HeLa cell mitochondrial lysates were treated with oxamate, and LDH activity was measured by using a colorimetric assay. b HeLa cell proliferation was measured after treatment with oxamate for 24 h. No DMSO was added to the samples. Data shown are averages from groups of n = 3. ***p < 0.001; n.s., no statistical significance. (PDF 60 kb) [file 40170_2018_192_MOESM4_ESM.pdf]

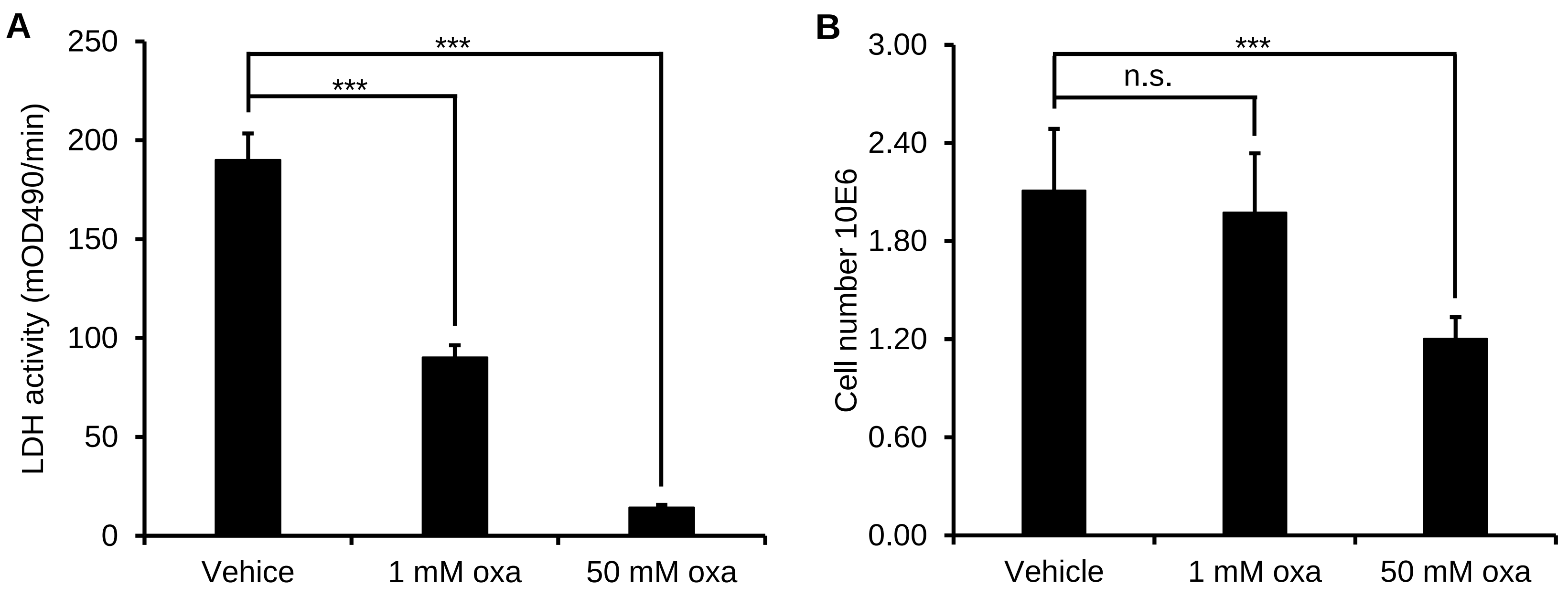

Figure S4: Response of HeLa cells to different concentrations of oxamate.
(A) HeLa cell mitochondrial lysates were treated with oxamate, and LDH activity was measured by using a colorimetric assay. (B) HeLa cell proliferation was measured after treatment with oxamate for 24 h. No DMSO was added to the samples. Data shown are averages from groups of n=3. ***$p < 0.001$; n.s., no statistical significance.
