# Supplementary material for: Transport-exclusion pharmacology to localize lactate dehydrogenase activity within cells
Source: Cancer Metab. 2018 Dec 12;6:19. doi: 10.1186/s40170-018-0192-5 (PMC6290536; doi:10.1186/s40170-018-0192-5)

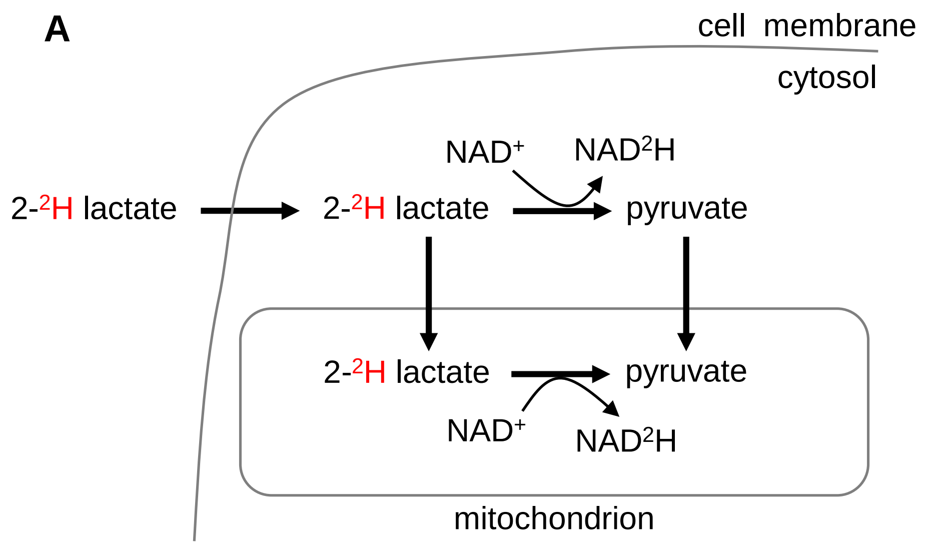

Figure S5: Deuterium labels from ^{2}H-lactate enter Hep G2 and H460 mitochondria. (A) Lactate can be exchanged with pyruvate in the cytosol or lactate can be transported into mitochondria. We sought to distinguish between these two possibilities by labeling cells with 2-^{2}H lactate. We then used LC/MS to localize 2-^{2}H lactate to mitochondria. (B-C) Mass spectra from mitochondria purified from cells cultured for 45 minutes in 2-^{2}H lactate enriched media. (B) Purified mitochondria from Hep G2, a human liver cancer cell line. (C) Purified mitochondria from H460, a human lung cancer cell line. These results support a pool of transported lactate in mitochondria.

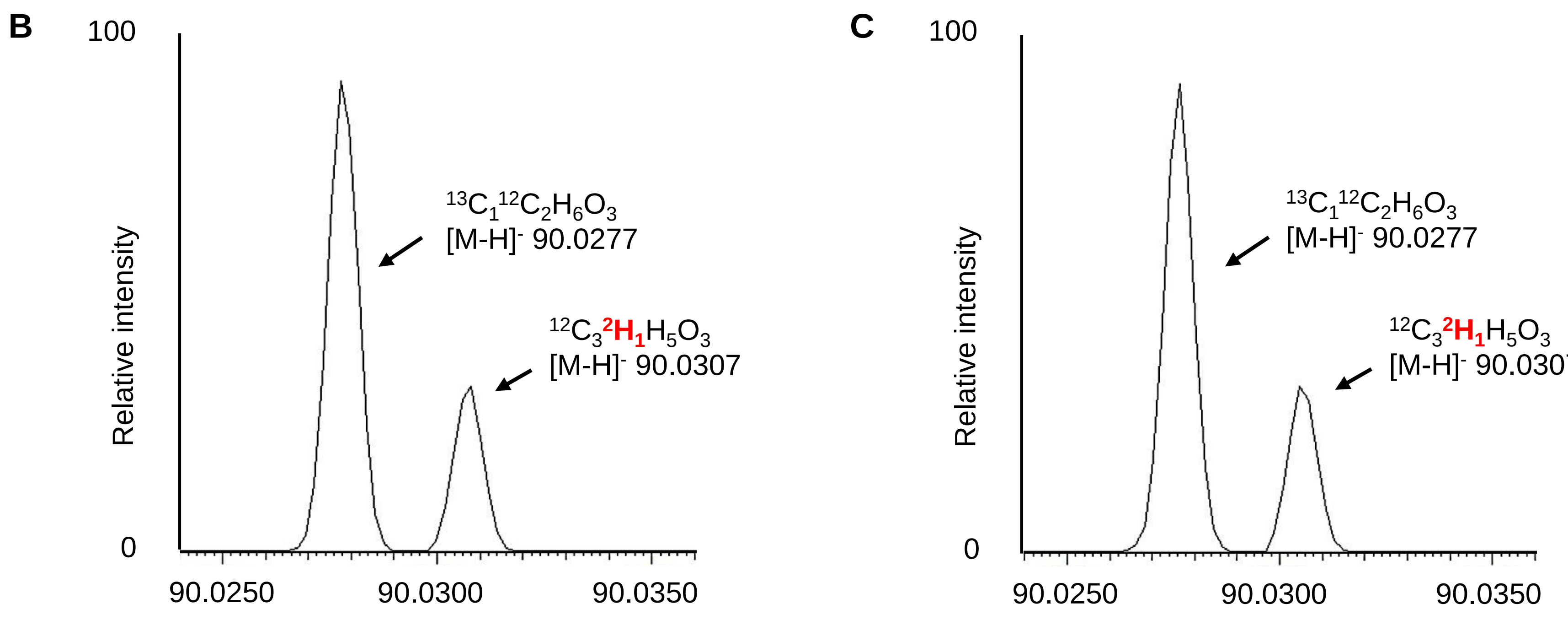

Supplement: Supplementary file 5 — Figure S5. Deuterium labels from 2-2H lactate enter Hep G2 and H460 mitochondria. a Lactate can be exchanged with pyruvate in the cytosol or lactate can be transported into mitochondria. We sought to distinguish between these two possibilities by labeling cells with 2-2H lactate. We then used LC/MS to localize 2-2H lactate to mitochondria. b, c Mass spectra from mitochondria purified from cells cultured for 45 min in 2-2H lactate enriched media. b Purified mitochondria from Hep G2, a human liver cancer cell line. c Purified mitochondria from H460, a human lung cancer cell line. These results support a pool of transported lactate in mitochondria. (PDF 145 kb) [file 40170_2018_192_MOESM5_ESM.pdf]
